# Clinical feasibility of a new full-thickness endoscopic plication device (GERDx™) for patients with GERD: results of a prospective trial

**DOI:** 10.1007/s00464-018-6153-9

**Published:** 2018-03-30

**Authors:** Michael Weitzendorfer, Georg O. Spaun, Stavros A. Antoniou, Kai Witzel, Klaus Emmanuel, Oliver O. Koch

**Affiliations:** 1 Department of Surgery, Paracelsus Medical University, 5020 Salzburg, Austria; 2 Department of Surgery, Ordensklinikum Linz Barmherzige Schwestern, Linz, Austria; 3 Department of General Surgery, 401 Military Hospital, Athens, Greece; 4 Minimal Invasiv Center, Hersfelder Strasse 1, 36088 Huenfeld, Germany

**Keywords:** GERD, Quality of life, Reflux activity, Endoscopic full-thickness plication

## Abstract

**Background:**

Previous studies suggest clinical effectiveness of endoscopic full-thickness plication in selected patients with gastroesophageal reflux disease (GERD). The aim of this study was to assess the clinical safety and efficiency of the GERDx™ device by evaluating clinical parameters, reflux symptom scores, and quality of life (QoL).

**Methods:**

Prospective one-arm trial evaluating the outcome of forty patients with GERD subjected to endoscopic plication with the GERDx™ device. We included patients with at least one typical reflux symptom despite treatment with a PPI for > 6 months, pathologic esophageal acid exposure, hiatal hernia of size < 2 cm, and endoscopic Hill grade II–III. Evaluation of Gastrointestinal Quality of Life Index (GIQLI), symptom scores, esophageal manometry, and impedance-pH-monitoring were performed at baseline and at 3 months after surgery. (Trial Registration: ClinicalTrials.gov NCT 01798212.)

**Results:**

There were no intraoperative complications. Four out of forty patients experienced postoperative complications requiring intervention. Seven of forty patients were subjected to laparoscopic fundoplication 3 months after endoscopic plication due to persistent symptoms and were lost to further follow-up. Thirty out of forty patients were available at 3-month follow-up. There was an improvement of the GIQLI score, from a mean of 92.45 ± 18.47 to 112.03 ± 13.11 (*p* < 0.001). The general reflux-specific score increased from a mean of 49.84 ± 24.83 to 23.93 ± 15.63 (*p* < 0.001), and the DeMeester score from a mean of 46.48 ± 30.83 to 20.03 ± 23.62 (*p* < 0.001). There was no significant change in manometric data after intervention. Three of thirty patients continued daily antireflux medication.

**Conclusions:**

Endoscopic plication with the GERDx™ device reduced distal acid exposure of the esophagus, reflux-related symptoms, and improved GIQLI scores with minimal side effects in a selected cohort of patients and may be a safe alternative in the treatment of GERD.

Daily use of proton pump inhibitors (PPI) is generally effective in the treatment of the majority of patients with gastroesophageal reflux disease (GERD); however, up to 40% have persisting symptoms [1, 2]. The primary alternative to chronic PPI medication is laparoscopic antireflux surgery (LARS). Although surgery generally results in excellent control of reflux symptoms in the long term, both surgeons and patients are frequently reluctant to proceed to select this option [3]. Indeed, LARS is associated with short- and long-term dysphagia, gas-bloat syndrome, and bowel dysfunction in a significant proportion of patients [4]. Hence, there is a broad desire to develop less invasive techniques without the limitations of pharmacologic therapy and the risks of LARS.

Flexible endoscopic techniques have been evaluated as treatment alternatives in the past; however, several had to be withdrawn from the market because of lack of effectiveness or due to safety concerns. There are currently four devices available for endoscopic treatment of GERD: a transoral incisionless fundoplication device (EsophyX®, EndoGastric Solutions, Redmond, WA), a radiofrequency energy delivery system (Stretta®, Mederi Therapeutics, Inc., Greenwich, CT), the Ultrasonic Surgical Endostapler device (MUSE™, Medigus, Omer, Israel), and GERDx™ (G-SURG GmbH, Seeon-Seebruck, Germany), a recently launched endoscopic plication device. The first short-term outcomes of GERDx™ suggest an improvement of objective reflux parameters and quality of life [5].

The objective of the present report is to present the short follow-up of a larger prospective cohort of patients subjected to endoscopic plication with the GERDx™ device, aiming at assessing its safety and efficacy in terms of symptom control and functional parameters.

## Materials and methods

### Study population

**Fig. 1:**
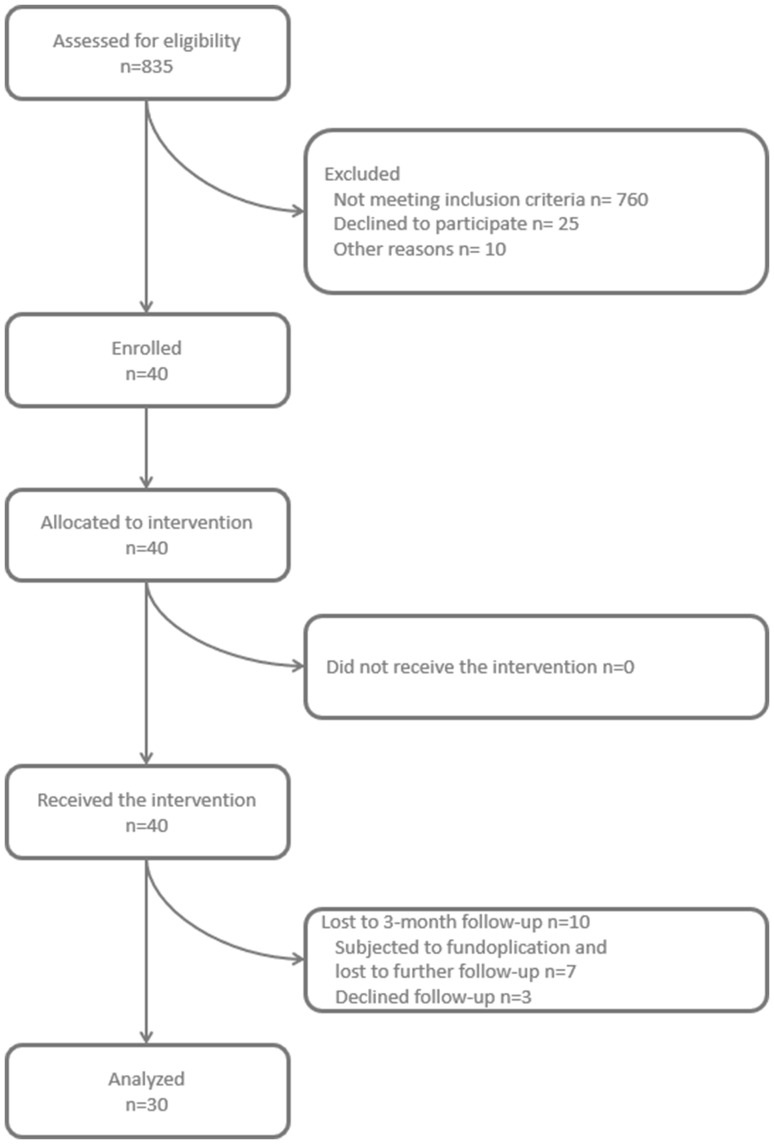
Consort

From October 2012 to December 2016, 835 individuals with symptoms of chronic GERD were assessed for eligibility at the Department of General and Visceral Surgery, Ordensklinikum Linz Sisters of Charity Hospital in Linz (Fig. 1). All patients were subjected to gastroscopy, barium esophagography, high-resolution esophageal manometry, and esophageal 24-h multichannel intraluminal impedance monitoring.

Inclusion criteria were as follows: at least one typical reflux symptom despite treatment with a PPI for at least 6 months and pathologic esophageal acid exposure as documented by a reflux-related DeMeester score of > 14.7, or symptom correlation (SI) > 50%, or > 73 reflux episodes per day. All subjects were candidates for LARS, according to the guidelines of the Society of American Gastrointestinal and Endoscopic Surgeons [6].

Exclusion criteria were as follows: age < 18 years, American Society of Anaesthesiologists physical status classification III-IV, evidence of a paraesophageal hernia or hiatal hernia measuring > 2 cm upon gastroscopy or barium esophagogram, gastroesophageal flap valve (GEFV) grade IV, previous esophageal or gastric surgery and pregnancy.

Study approval was obtained by the institution’s ethical committee, and all patients provided written informed consent.

The study was registered at ClinicalTrials.gov (Identifier: NCT 01798212).

### Study design and follow-up

Prospective single-center one-arm trial on the clinical and functional outcomes of endoscopic plication with GERDx™. The GIQLI and symptom scores were calculated at baseline. Gastroscopy, high-resolution esophageal manometry, and 24-h pH-metry-impedance were performed before the intervention. The patients received a PPI treatment on daily basis for 2 weeks after plication. A gastroscopy was performed 6 weeks after the procedure and the GIQLI and symptom scores were calculated at 3-month follow-up, along with functional assessment through manometry and 24-h pH-metry-impedance.

The primary outcome measure was difference in the GIQLI after the intervention of at least 15 points. Secondary endpoints were difference in esophageal acid exposure, reflux-specific symptom scores, and perioperative morbidity.

### Endoscopic full-thickness plication technique

**Fig. 2:**
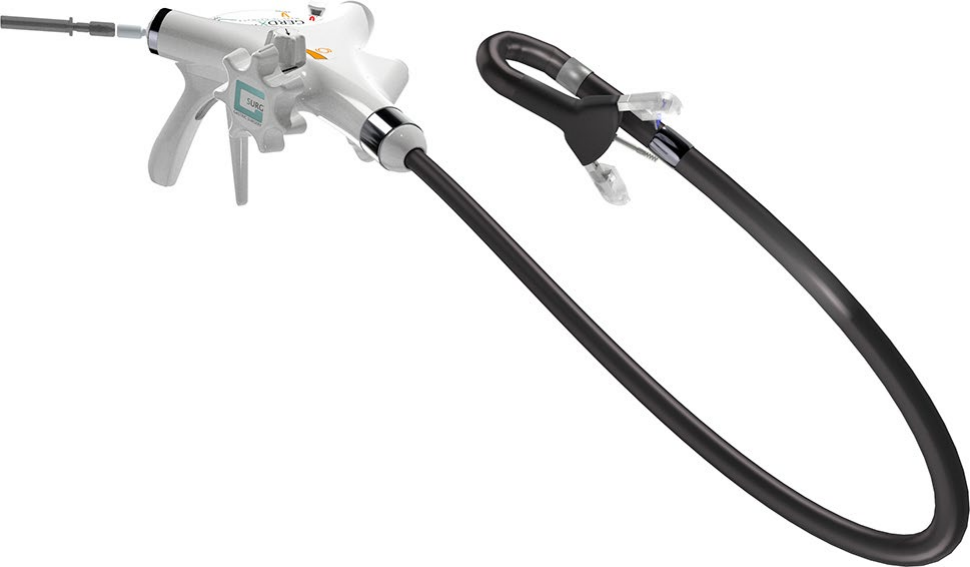
GERDx™ (G-SURG GmbH, Seeon-Seebruck, Germany)

Endoscopic full-thickness plication was performed using the GERDx™—system (G-SURG GmbH, Seeon-Seebruck, Germany). The GERDx™—device uses hydraulic elements for controlling and is the advanced single use product of a company that has taken over the Plicator technology, after the Plicator device (Ethicon Endosurgery, Sommerville, NJ) was taken off the market (Fig. 2). The design modifications of GERDx™ do not hinder clinical application and it shows similar safety in application compared to the NDO Plicator [5].

All procedures were performed under general anesthesia by the same surgical team with established experience in advanced endoscopic procedures. No peri-procedure antibiotics were given. A standard upper endoscopy was performed and a Savary-guidewire was placed upon gastroscope withdrawal. The GERDx™ device was introduced over the guidewire and into the stomach.

A 5.8-mm video endoscope (N-190 Olympus, Tokyo, Japan) was passed through the GERDx™ device. The distal end of the device was then retroflexed to the anterior gastric cardia approximately 1 cm below the gastroesophageal (GE) junction. The GERDx™ arms were opened, and an endoscopic tissue retractor was advanced deeply into the gastric cardia. The tissue retractor was drawn back to gather tissue between the open arms of the GERDx™ device. The arms were closed and a pre-tied transmural pledgeted suture was deployed. At the end of procedure, the GERDx™ device and the gastroscope were removed, and the gastroscope was re-inserted to evaluate the resulting plication.

**Fig. 3:**
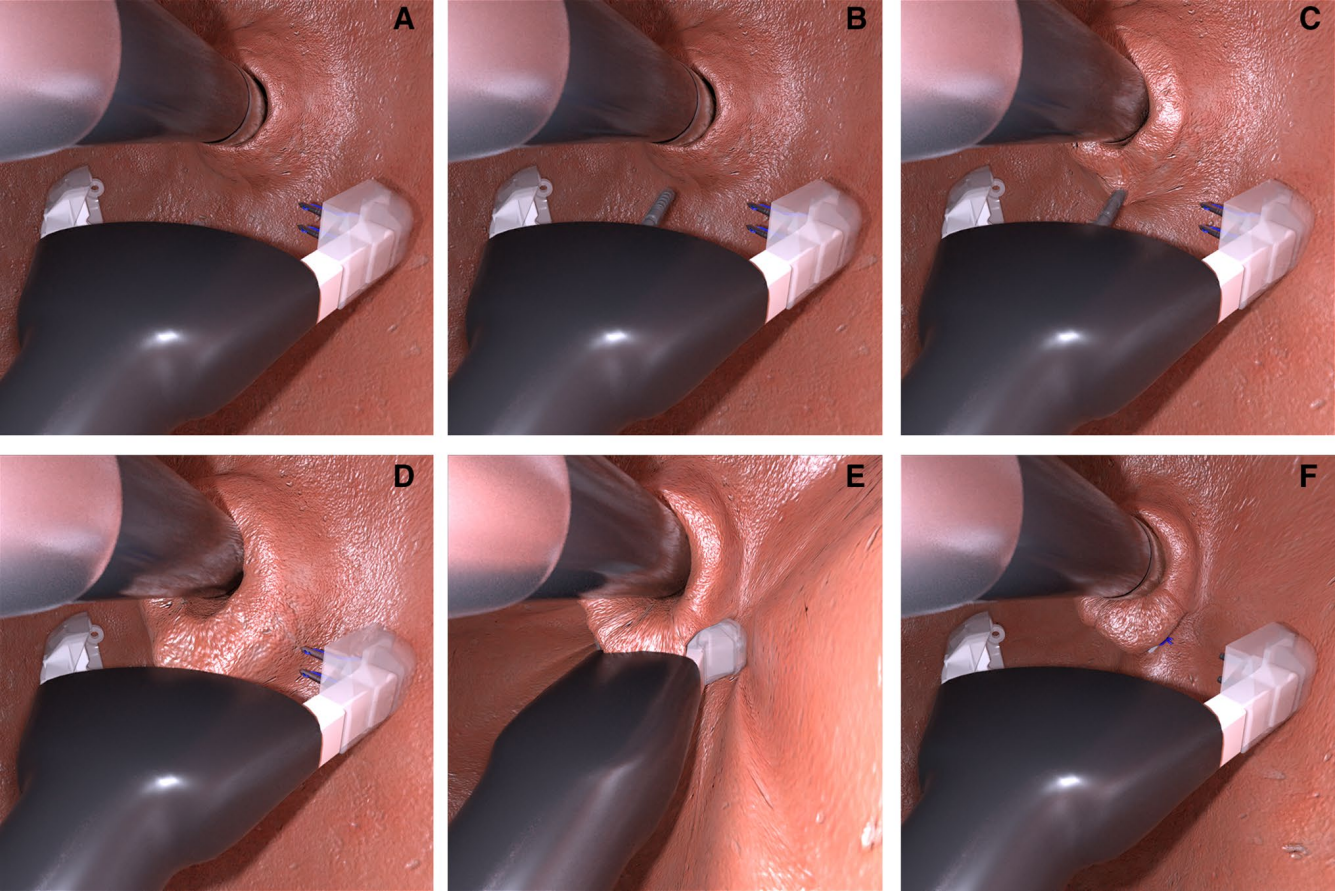
**A** GERDx™ arms are opened. **B** Tissue retractor is advanced to serosa. **C, D** Gastric wall is retracted into the GERDx™ arms. **E** A pre-tied transmural pledgeted suture is deployed. **F** Full-thickness plication is restructuring the GE junction

According to study protocol, at least two pre-tied transmural pledgeted sutures were deployed. One or two additional sutures were placed, if necessary, until a tight closure of the gastroesophageal junction around the endoscope was achieved (Fig. 3).

### Quality of life evaluation (QoL)

Quality of life was evaluated by means of the German gastrointestinal quality of life index (GIQLI) [7]. This questionnaire has been validated in the German language and has been recommended for use by the European Study Group for Antireflux Surgery [8]. The GIQLI is divided into 5 domains and 36 items: gastrointestinal symptoms (0–76 points), physical functions (0–28 points), emotional status (0–20 points), social functions (0–16 points), and a single item for stress of medical treatment (0–4 points), for a minimum of 0 and a maximum of 144 points. Higher scores indicate a better quality of life. Information on daily or on-demand use of PPI or other antacid medication was obtained.

### Symptom evaluation

Symptom evaluation was carried out in a standardized way using a written questionnaire assessing the severity and intensity of 14 symptoms in a 4-point scale (SCL). This questionnaire has been used previously in the context of GERD [9]. Symptoms of heartburn, chest pain, regurgitation, hoarseness, cough, asthma, dysphagia, fullness, diarrhea, flatulence, constipation, belching, bloatedness, and distortion of taste are graded as none (0), once per week (1), several times per week (2), daily (3), and constantly (4). Intensity of the symptoms is graded as none (0), mild (1), moderate (2), severe (3), and extremely severe (4). In order to obtain the ultimate result, the frequency of each symptom is multiplied by its degree, resulting in scores from 0 to 16 for each symptom, for a total maximum score of 224 and a minimum score of 0 points. Additionally, four different scores were extracted to assess symptoms specific for reflux (heartburn, regurgitation, chest pain), gas-bloat (fullness, bloatedness), bowel-dysfunction (diarrhea, constipation, flatulence), and atypical reflux symptoms (cough, hoarseness, asthma, distortion of taste). Symptoms of dysphagia and belching are evaluated separately.

### High-resolution esophageal manometry (HRM)

All patients were studied after an overnight fast in the supine position. A high-resolution manometry (HRM) using the Sierra system (Given Imaging, Duluth, GA, USA) was performed. A structurally defective lower esophageal sphincter (LES) was defined as an overall length below 2.4 cm, an intraabdominal length below 0.9 cm and/or the presence of a hiatal hernia. Pressure levels beyond the range of 29.8–180.2 mmHg were considered abnormal and any motility disorders were classified according to the Chicago Classification [10].

### 24-h ambulatory multichannel intraluminal impedance monitoring (MII)

Studies were performed after cessation of antisecretory therapy for at least 7 days. A catheter-based 24-h multichannel intraluminal impedance pH-metry (pH/MII, Dual-probe; Given Imaging, Duluth, GA, USA) was inserted. Further details have been published previously [9].

Symptom Index (SI) of > 50% was considered positive [11].

GERD was defined as an abnormal esophageal acid exposure, total number of reflux events within 24 h > 73, DeMeester score > 14.7, or positive SI for symptoms reported at least three times. In addition, elevated counts of acidic, weakly acidic, and non-acidic reflux episodes demonstrated by the impedance signal of the pH-catheter were considered abnormal [12, 13].

### Statistical analysis

Statistical analysis was performed using SPSS-Statistical-Analysis Software (SPSS Inc., Chicago, IL, USA). Sample size considerations based on the assumption of a one-sided test (Wilcoxon signed-rank test) with *α* = 0.025 and power 1−β = 0.9 with respect to a medically relevant (absolute) effect size of 15 points in the change of the GIQLI-index suggested enrolment of at least 40 patients. All datasets were tested for normal distribution by Kolmogorov–Smirnow-Test. Data were compared using two-tailed paired-*t* test or Wilcoxon signed rank test, as applicable, on a per subject basis. Homogeneity of population was conducted using independent *t* test or Mann–Whitney *U* Test. Datasets were additionally presented as means and standard deviation (SD), if normally distributed. Previously published studies provided helpful information for variance estimation [5]. A *p* value below 0.05 was considered statistically significant.

## Results

Forty consecutive patients were enrolled in the study (Fig. 1). There were 18 male and 22 female patients with a mean age of 49.75 (± 13.8) years and a mean BMI of 24.85 (± 3.6) kg/m^2^.

The mean procedural time was 34 ± 10.5 min. Twenty-five of forty patients received primarily two GERDx™—implants; fourteen received three and in another patient four GERDx™—implants were deployed. All forty procedures were performed by the same surgical team with established experience in advanced endoscopic procedures (G.O.S. and O.O.K.).

Seven out of forty patients (17.5%) underwent LARS before the 3-month follow-up, six due to persistent symptoms and one due a postoperative complication after endoscopic plication with GERDx™. In this patient, the gastric fundus had to be oversewn due to leakage after the release of adhesions. The other six patients presented with typical and/or atypical GERD-related symptoms at the time of control gastroscopy 6 weeks after endoscopic plication. All six patients had persistent signs of esophagitis and three out of these six patients (50.0%) showed disrupted sutures. In addition, all patients showed pathological results in pH measurement and were considered to undergo laparoscopic fundoplication. Furthermore, in all patients, a small hiatal hernia was found during subsequent LARS, which was considered as reason for failure beside the disrupted sutures after plication.

Three out of forty patients (7.5%) did not wish to further participate in the study. In total, 30 (75%) patients were available at 3-month follow-up.

### 24-h-pH-metry-impedance (MII)

Mean DeMeester score was reduced from 46.48 ± 30.83 to 20.03 ± 23.62 at follow-up (Table 1). Furthermore, in 18 out of 30 patients (60.0%), the DeMeester score was within normal levels (< 14.72). Five out of these eighteen patients (27.8%) were on PPIs.

**Table 1:** DeMeester score, GIQLI, and general reflux-specific symptom scores at baseline vs. 3-month follow-up

	De Meester score		GIQLI		SCL	
	Baseline *n* = 30	3 months *n* = 30	Baseline *n* = 30	3 months *n* = 30	Baseline *n* = 30	3 months *n* = 30
Mean	46.48	20.03	92.45	112.03	49.84	23.93
Standard deviation	30.83	23.62	18.47	13.11	24.83	15.63
Median	41.35	13.20	92.50	114.00	47.00	20.00
Minimum	7.80	1.00	32.00	84.00	12.00	0.00
Maximum	139.60	93.50	124.00	139.00	137.00	75.00
Significance	*p* < 0.001 (*p* = 0.000)		*p* < 0.001 (*p* = 0.000)		*p* < 0.001 (*p* = 0.000)	

*GIQLI* (mean normal 122.6) gastrointestinal quality of life index, *SCL* summarization of typical reflux-, atypical reflux-, gas/bloating-, bowel dysfunction-, dysphagia-, and belching scores

The mean number of total, acid and weakly acid reflux episodes were reduced after the procedure. No nonacid reflux episodes have been detected before or after procedure (Table 2). In addition, nine out of 12 patients (75.0%), who did not achieve pH normalization, had a Hill Grade III valve before the procedure. Thirteen out of 18 patients (72.2%), who showed normal results in pH measurement after the procedure, also had a Hill Grade III valve (*p* = 0.60; odds ratio 1.15, 95% confidence interval 0.22–6.10).

**Table 2:** Reflux episodes detected by MII-pH-monitoring at baseline vs. 3-month follow-up

	Reflux episodes in total		Reflux episodes acidic		Reflux episodes weakly acidic	
	Baseline *n* = 30	3 months *n* = 30	Baseline *n* = 30	3 months *n* = 30	Baseline *n* = 30	3 months *n* = 30
Mean	148.42	69.59	129.08	56.86	15.21	12.72
Standard deviation	108.91	63.87	111.41	56.94	22.06	23.06
Median	122.50	42.00	107.50	35.00	8.00	5.00
Minimum	16.00	0.00	7.00	0.00	0.00	0.00
Maximum	563.00	245.00	563.00	220.00	117.00	117.00
Significance	*p* < 0.01 (*p* = 0.001)		*p* < 0.01 (*p* = 0.007)		No significance (*p* = 0.160)	

### Gastroscopy

In gastroscopy, 26 of 40 patients (65.0%) had signs of esophagitis (grade I or II) before the intervention. All seven patients who had to be re-operated on had esophagitis at preoperative esophagogastroscopy. Of the 30 patients available at follow-up, 6 of 30 (20%) had esophagitis (*p* < 0.05); grade II esophagitis or higher was not found in any patient. All patients were off PPI at least 1 week before gastroscopy.

### High-resolution esophageal manometry (HRM)

LES-resting-pressure (LESP) improved from 22.60 ± 10.96 mmHg at baseline to 23.17 ± 12.47 mmHg at 3-month follow-up, without statistical significance (*p* = 0.779). The procedure had no significant impact on esophageal body motility. Furthermore, all patients showed normal values of the upper esophageal sphincter pressure (UESP), integrated relaxation pressure (IRP) as well as distal contractile integral (DCI) before and after the procedure.

### Quality of life (QoL)

The baseline mean general GIQLI was 92.45 ± 18.47 points, which is significantly lower compared to healthy individuals (122.6 ± 8.5 points, *p* < 0.01) [15]. The mean GIQLI after the procedure increased to 112.03 ± 13.11 points (*p* < 0.001) (Table 1). Furthermore, all thirty patients who completed the 3-month follow-up showed an improvement of the GIQLI score after endoscopic full-thickness plication with the GERDx™ device.

### Symptom scores (SCL)

Mean general reflux-specific symptom score (SCL) was significantly reduced at 3-month follow-up (*p* < 0.001) (Table 1). Twenty-eight out of thirty patients (93.3%) showed an improvement of SCL after the procedure. Scores for typical reflux symptoms, bowel dysfunction, atypical reflux symptoms and gas/bloating scores all improved significantly (Table 3). In addition, the mean dysphagia score improved from 3.81 ± 4.69 to 0.83 ± 2.32 (*p* < 0.01) and the belching score decreased from 5.22 ± 4.47 to 3.36 ± 3.25 (*p* = 0.071).

**Table 3:** Reflux-specific symptom scores at baseline vs. 3-month follow-up

	Typical reflux		Atypical reflux		Bowel dysfunction		Gas/bloating	
	Baseline *n* = 30	3 months *n* = 30	Baseline *n* = 30	3 months *n* = 30	Baseline *n* = 30	3 months *n* = 30	Baseline *n* = 30	3 months *n* = 30
Mean	16.68	6.79	8.62	3.14	7.14	4.72	9.16	5.34
Standard deviation	9.87	6.94	8.82	4.66	5.73	3.78	7.04	4.61
Median	16.00	6.00	7.00	2.00	6.00	4.00	8.00	5.00
Minimum	1.00	0.00	0.00	0.00	0.00	0.00	0.00	0.00
Maximum	48.00	32.00	37.00	19.00	21.00	14.00	28.00	18.00
Significance	*p* < 0.001 (*p* = 0.000)		*p* < 0.01 (*p* = 0.001)		*p* < 0.001 (*p* = 0.000)		*p* < 0.01 (*p* = 0.001)	

### Use of anti-refluxmedication

Three out of 30 patients (10.0%) stated that they were on PPI medication on a daily basis, 8 of 30 patients (26.7%) on demand, and 19 of 30 (63.3%) were off medication after plication.

### Safety results and side effects

At 3-month follow-up, no residual serious adverse events (SAEs) related to the device or procedure were observed. All patients underwent the procedure without any intraoperative complication.

The most common adverse events (AEs) reported were sore throat in 8/40 patients (20%) and chest pain in 7/40 patients (17.5%). All reported AEs resolved spontaneously in the immediate post-operative period.

Four out of forty patients (10%) enrolled in the study had postoperative SAEs, which required intervention (https://www.fda.gov). Two SAEs were rated as moderate. The first involved a subject, who suffered from dysphagia and postoperative pain. A hematoma at the GE junction was diagnosed in computerized tomography (CT scan). The patient required a higher dose of pain medication postoperatively and the hematoma was reabsorbed at follow-up without further intervention. Another patient developed pneumonia with pleural effusion and required treatment with antibiotics. After 10 days, recovery was complete.

Two severe SAEs were recorded. The first severe SAE included a patient who showed laboratory evidence of severe inflammation and suffered from intractable postoperative pain. After CT-scan, a diagnostic laparoscopy had to be performed. During laparoscopy, a pledgeted suture passing through the left crus of diaphragm and left hepatic lobe had been detected. The suture had to be removed and the stomach oversewn and covered by a Dor fundoplication. 1 day postoperatively, the patient required placement of a chest tube, due to severe pleural effusion. 3 days later, a video-assisted thoracoscopic surgery (VATS) and decortication of a pleural empyema had to be performed. During VATS, a pledget in the pleural cavity was found as presumable cause of symptoms. At 3-month follow-up, the patient had a normal result in 24-h pH-monitoring and did not show consequential damages. The second severe SAE was a Mallory-Weiss-lesion at the GE junction. A gastroscopy was performed 1 day after primary surgery, because the patient suffered from intense postoperative pain. A 2-cm lesion was detected and treated by endoscopic clip application and the patient received further treatment with antibiotics. After another control gastroscopy 3 days later, the patient left hospital completely recovered. Summarizing data are provided at Table 4.

**Table 4:** Summary of procedure-related SAEs

SAE	Length of hospital stay (days)	Rating	Life threatening	SAE description	Readmission	Re-operation
1	8	Moderate	No	Hematoma at GE junction	No	No
2	10	Moderate	No	Pneumonia	No	No
3	29	Severe	No	Suture passing left hepatic lobe and pleural empyema	No	Yes
4	5	Severe	No	Mallory–Weiss–lesion at GE junction	No	No

*SAE* serious adverse event

There was no evidence of association of these adverse events with the learning curve, as the first 15 procedures were uneventful. Some adverse events (3 out of 6) and treatment failures (2 out of 6) might have been related however to a change of the suture material (2.0 monofilament non-absorbable suture to 0.0 braided non-absorbable suture) and suture length (6–7.6 mm) introduced by the company. The interim review of these early SAEs resulted in a device change and the next nineteen patients were treated with the original suture material and length again, with only one subsequent SAE [6]. Analysis without those 6 six patients showed no significant difference in objective and subjective measurements (data not shown).

## Discussion

The technique of endoscopic full-thickness plication has shown to be well tolerated and seems to improve the acid exposure of the distal esophagus, GERD symptoms, as well as QoL, usually without serious adverse events (SAEs) after surgery [9, 14]. Hence, there could be a future market for endoscopic full-thickness plication in well-selected patients.

The results of the reported trial with the new GERDx™ device seem to underline this hypothesis.

Endoscopic full-thickness plication with the GERDx™ device resulted in excellent symptom control in twenty-eight of thirty patients (93.3%). The amount of total reflux episodes, acid reflux episodes, and weakly acid reflux episodes decreased and a significant reduction of DeMeester score was found. However, only 60% of the patients experienced a normalization of DeMeester score, although all patients (100%) reported a better quality of life after surgery. The fact that not all of patients show a normalization of the distal acid exposure is something that the procedure shares with other endoscopic procedures like MUSE™ or Esophyx™ [15, 16].

Hill grade might be associated with treatment effect; however, we did not find significant difference between different grades, although this may be due to a type II error. Long-term data might elucidate this putative association.

Nevertheless, the significantly reduced rate of patients suffering from esophagitis after the procedure with the GERDx™ device (65–20%) suggests an adequate control of esophageal acid exposure. Furthermore, only 10% of the patients needed a PPI treatment on daily basis and 26.7% on demand at 3-month follow-up, which is comparable to other endoscopic techniques [15, 16].

Similarly to previous studies endoscopic plication did not have a significant impact on manometric characteristics [9]. This is in accordance to the theoretical framework, suggesting that both the LES and the diaphragm contribute to gastroesophageal sphincter competence and the procedure does not induce structural changes to the esophageal hiatus [17, 18].

The results of our study show a significant improvement of patient’s life quality assessed by the Gastrointestinal quality of life index (GIQLI) at 3-month follow-up, which is also comparable to procedures like MUSE™ or Esophyx™ [15, 16].

These data illustrate that the procedure can relieve heartburn symptoms and provide an effective alternative to chronic PPI use. Furthermore, reflux-specific symptom scores, (typical reflux-, and atypical reflux symptom scores) significantly improved after endoscopic full-thickness plication, and the patients showed no side effects like bowel dysfunction, gas/bloating, or dysphagia.

In addition, the relatively low mean procedural time compared to other endoscopic procedures or laparoscopic fundoplication could be a benefit of the GERDx™ device, considering aspects of cost-effectiveness [15, 19].

Adverse events in this cohort might be related to the suture characteristics temporarily introduced by the company, as was previously reported [5]. Although the safety profile of the device requires further evaluation, current data suggest that endoscopic plication is a safe procedure in experienced hands. The protocol of this study required hospitalization and observation for all patients to ensure patient safety. However, the procedure of endoscopic full-thickness plication may eventually be performed in an outpatient setting as experience increases.

This is a single-arm study, which limits our confidence on treatment effect estimates. Randomized controlled trials are necessary to compare the outcome of patients treated with PPIs and patients undergoing endoscopic plication with the GERDx™ device. Paired analyses may provide robust results; however, they cannot eliminate the possibility of placebo effect.

Furthermore, power calculations were based on the primary outcome measure related to the quality of life. The 25% lost of follow-up-rate is a factor, which potentially affects the validity of the results and the study’s power calculation. The study might be therefore not adequately powered to demonstrate significance in treatment outcomes for the secondary parameters. Longer duration follow-up is pending. However, the report of these early results is important since the device is commercially available.

The outcomes of this study must be observed in the context of the study population. We included only patients with hiatal hernia measuring < 2 cm and excluded individuals with Barrett’s esophagus or esophageal motility disorders. We consider reasonable not to recommend this procedure to such patients until further studies assess its safety and efficacy. The learning curve of the procedure could be limiting factor for a widespread application, because high operator expertise is needed to achieve a good procedure outcome. It needs to be discussed, if a preoperative computed tomography (CT) scan to measure the hiatal surface area could be a screening option to increase the procedure outcome by the exclusion of unsuitable patients.

In conclusion, endoscopic full-thickness plication using the GERDx™ device improves the distal acid exposure of the esophagus, typical reflux-related symptoms and QoL in well-selected patients. This procedure might constitute an option for patients with mild GERD. Long-term outcomes are required to expand our knowledge on the effects of this procedure.
